# The multidrug resistance transporter P-glycoprotein confers resistance to ferroptosis inducers

**DOI:** 10.20517/cdr.2023.29

**Published:** 2023-07-27

**Authors:** William J. E. Frye, Lyn M. Huff, José M. González Dalmasy, Paula Salazar, Rachel M. Carter, Ryan T. Gensler, Dominic Esposito, Robert W. Robey, Suresh V. Ambudkar, Michael M. Gottesman

**Affiliations:** ^1^Laboratory of Cell Biology, Center for Cancer Research, National Cancer Institute, National Institutes of Health, Bethesda, MD 20892, USA.; ^2^Protein Expression Laboratory, Frederick National Laboratory for Cancer Research, Leidos Biomedical Research, Inc, Frederick, MD 21704, USA.; ^#^These authors contributed equally to this work.

**Keywords:** Ferroptosis, drug resistance, P-glycoprotein, ABCG2

## Abstract

**Aim:** Ferroptosis is a non-apoptotic form of cell death caused by lethal lipid peroxidation. Several small molecule ferroptosis inducers (FINs) have been reported, yet little information is available regarding their interaction with the ATP-binding cassette (ABC) transporters P-glycoprotein (P-gp, ABCB1) and ABCG2. We thus sought to characterize the interactions of FINs with P-gp and ABCG2, which may provide information regarding oral bioavailability and brain penetration and predict drug-drug interactions.

**Methods:** Cytotoxicity assays with ferroptosis-sensitive A673 cells transfected to express P-gp or ABCG2 were used to determine the ability of the transporters to confer resistance to FINs; confirmatory studies were performed in OVCAR8 and NCI/ADR-RES cells. The ability of FINs to inhibit P-gp or ABCG2 was determined using the fluorescent substrates rhodamine 123 or purpuin-18, respectively.

**Results:** P-gp overexpression conferred resistance to FIN56 and the erastin derivatives imidazole ketone erastin and piperazine erastin. P-gp-mediated resistance to imidazole ketone erastin and piperazine erastin was also reversed in UO-31 renal cancer cells by CRISPR-mediated knockout of *ABCB1*. The FINs ML-162, GPX inhibitor 26a, and PACMA31 at 10 µM were able to increase intracellular rhodamine 123 fluorescence over 10-fold in P-gp-expressing MDR-19 cells. GPX inhibitor 26a was able to increase intracellular purpurin-18 fluorescence over 4-fold in ABCG2-expressing R-5 cells.

**Conclusion:** Expression of P-gp may reduce the efficacy of these FINs in cancers that express the transporter and may prevent access to sanctuary sites such as the brain. The ability of some FINs to inhibit P-gp and ABCG2 suggests potential drug-drug interactions.

## INTRODUCTION

Ferroptosis is an iron-dependent form of non-apoptotic cell death arising from direct or indirect inhibition of glutathione peroxidase 4 (GPX4), leading to lipid peroxidation and unsustainable levels of reactive oxygen species (ROS)^[1]^. One of the first reported inducers of ferroptosis, erastin, was described before the concept of ferroptosis was completely understood. Erastin was found to selectively kill transformed human foreskin fibroblasts expressing mutant HRAS compared to isogenic cells expressing wild-type HRAS^[2]^. The mechanism of cell death induced by erastin was not apoptosis, as the hallmarks of apoptotic cell death such as annexin V staining and caspase 3 cleavage were not observed, although death was accompanied by cell membrane permeabilization^[2]^. The term “ferroptosis” was later coined for this novel form of cell death as iron chelators and antioxidants were found to potentiate erastin-mediated toxicity, suggesting an iron-dependent increase in ROS was responsible^[3]^. Additionally, ferroptosis could not be inhibited by caspase inhibitors and was found to occur independently of the apoptosis effector proteins Bak and Bax^[3]^.

The target of erastin was identified to be the cystine/glutamate antiporter, system x^-^_c_^[3]^. Inhibition of the antiporter leads to depletion of glutathione and inactivation of GPX4^[4]^. Subsequent to the discovery of erastin, other small molecules have been developed to induce ferroptosis by direct or indirect inhibition of GPX4. These ferroptosis inducers (FINs) include modified forms of erastin such as erastin2^[5]^, imidazole ketone erastin^[6]^ and piperazine erastin^[4]^, as well as the inhibitors FIN56^[7]^, RSL3^[8]^, FINO_2_^[9]^, PACMA31^[10]^, GPX4 inhibitor 26a^[11]^, and ML-162 and ML-210^[12]^.

In an attempt to identify cancers that might be effectively treated by ferroptosis induction, Yang *et al*. tested erastin toxicity in 117 cancer cell lines and identified renal cell carcinomas as being particularly sensitive to ferroptosis^[4]^. This intrigued us, as renal cell carcinomas are often positive for expression of P-glycoprotein (P-gp, encoded by the *ABCB1* gene), an ATP-binding cassette (ABC) multidrug efflux pump that confers drug resistance^[13,14]^. Some reports have also suggested that expression levels of another ABC transporter, ABCG2 (encoded by the *ABCG2* gene), also expressed in kidney cancers, can predict overall survival in patients with clear cell renal carcinoma^[15]^. Additionally, both P-gp and ABCG2 localize to the gastrointestinal tract as well as to other barrier sites, such as the blood-brain barrier (BBB)^[16]^. Expression at these sites is linked to their role in limiting the oral bioavailability of chemotherapy drugs and brain penetration of several targeted therapies^[16,17]^. We thus sought to characterize the interactions between small-molecule ferroptosis inducers and the transporters P-gp and ABCG2.

## METHODS

### Chemicals

Erastin, doxorubicin, rhodamine 123, and FIN56 were purchased from Sigma-Aldrich (St. Louis, MO). Erastin2, imidazole ketone erastin, RSL3, ML-162, FINO_2_, GPX4 inhibitor 26a, JKE-1674, and JKE-1716 were from Cayman Chemical (Ann Arbor, MI). Valspodar was obtained from MedChemExpress (Monmouth Junction, NJ). Romidepsin was from Selleck Chemicals (Houston, TX). Purpurin-18 was purchased from Frontier Scientific (Logan, UT). Piperazine erastin was from TargetMol (Wellesly Hills, MA). SN-38 was obtained from LKT Laboratories (St. Paul, MN). RSL3 was purchased from Tocris (Minneapolis, MN). Fumitremorgin C (FTC, > 95% purity) was synthesized in-house by the Developmental Therapeutics Program at the National Institutes of Health (Bethesda, MD).

### Cell lines

OVCAR8, NCI/ADR-RES and UO-31 cells were obtained from the Division of Cancer Treatment and Diagnosis Tumor Repository, National Cancer Institute (Frederick, MD) and are grown in RPMI-1640 with 10% FBS, glutamine and Pen/Strep. A673 cells (from ATCC, Manassas, VA) were seeded and transfected with empty vector (EV) or vector containing full-length, human *ABCB1* or *ABCG2* using Lipofectamine 2000 (Invitrogen, Waltham, MA). Cells were selected with hygromycin, and clones were isolated by limiting dilution. Selected clones were grown in DMEM with 10% fetal calf serum, glutamine and Pen/Strep, as well as 300 µg/mL hygromycin to maintain expression of the transporters. P-gp-overexpressing MDR-19 cells and ABCG2-overexpressing R-5 cells were derived from HEK293 cells and have been previously characterized and described^[18]^. Transfected HEK293 cells were grown in MEM with 10% fetal calf serum, glutamine and Pen/Strep along with 2 mg/mL G418 to maintain expression of the transporters. All cell lines were routinely tested for mycoplasma using the MycoAlert PLUS Kit (Promega, Madison, WI) test kit and their identities were confirmed by STR analysis (performed by ATCC, Manassas, VA).

### Oligonucleotides

The following oligonucleotides (generated by Eurofins, Inc, Louisville, KY) were used in this study:

ABCB1-START: 5’- GGGGACAACTTTGTACAAAAAAGTTGGCACCATGGATCTTGAAGGGGACCGCAATGG

ABCB1-END: 5’- GGGGACAACTTTGTACAAGAAAGTTGATTATGCTAGCTGGCGCTTTGTTCCAGCCTGG

ABCG2-START: 5’- GGGGACAACTTTGTACAAAAAAGTTGGCACCATGTCTTCCAGTAATGTCGAAGTTTTTATCCC

ABCG2-END: 5’- GGGGACAACTTTGTACAAGAAAGTTGATTAAGAATACTTTTTAAGAAATAACAATTTCAG

### Generation of entry clones

Entry clones for *ABCB1* and *ABCG2* were constructed by PCR amplification of cDNA sequences flanked by Gateway Multisite recombination sites (Thermo Fisher Scientific, Waltham, MA). PCR was carried out with 200 nM of each oligo listed in the table above using Phusion polymerase (New England Biolabs, Ipswich, MA) under standard conditions and an extension time of 180 s. PCR products were cleaned using the QiaQuick PCR purification kit (Qiagen, Germantown, MD). The final PCR products were recombined into Gateway Donor vector pDonr-253 using the Gateway BP recombination reaction using the manufacturer’s protocols. The subsequent Entry clones were sequence verified throughout the entire cloned region.

### Subcloning for mammalian expression constructs

Gateway Multisite LR recombination was used to construct the final mammalian expression constructs from the Entry clones using the manufacturer’s protocols (Thermo Fisher Scientific, Waltham, MA). The Gateway Destination vector used was pDest-305 (Addgene. Watertown, MA, #161895), a mammalian expression vector containing a Gateway attR4-attR2 cassette based on a modified version of pcDNA3.1. This vector backbone contains a hygromycin resistance marker for antibiotic selection. A human elongation factor 1 (EF1) promoter was introduced using a Gateway att4-att1 Entry clone (Addgene, Watertown, MA, #162920). Final expression clones were verified by restriction analysis and maxiprep DNA was prepared using the Qiaprep Maxiprep kit (Qiagen, Germantown, MD).

### Generation of ABCB1 knockout UO-31 cells

CRISPR-mediated knockout of *ABCB1* in UO-31 cells was achieved by co-transfecting cells with knockout and homology-directed repair vectors for *ABCB1* (obtained from Santa Cruz Biotechnology, Dallas, TX) using Lipofectamine 2000 (Invitrogen) and subsequent selection with puromycin (3 µg/mL). Knockout clones were subsequently isolated, and loss of P-gp was verified by flow cytometry following antibody staining with phycoerythrin-labeled UIC-2 antibody as described below.

### Cytotoxicity assays

Cells were seeded in opaque white, 96-well plates at a density of 2,500 cells/well and allowed to attach overnight. Cells were then treated with increasing concentrations of the desired compound and incubated for 72 h. Cell TiterGlo (Promega) was then used to determine luminescence values for each concentration according to the manufacturer’s instructions. The data were modeled using nonlinear regression curve fitting (sigmoidal, 4 parameter logistic curve model in GraphPad Prism 9 for MacOS v 9.5.1, GraphPad Software, Boston, MA) to determine the concentration at which 50% of cell growth was inhibited (GI_50_). Where noted, cytotoxicity assays were performed with 10 µM valspodar to inhibit P-gp.

### Flow cytometry assays

To measure cell surface expression of P-gp or ABCG2, trypsinized cells were incubated for 20 min. at room temperature in 2% bovine serum albumin/PBS with phycoerythrin-labeled UIC-2 antibody or phycoerythrin-labeled 5D3 antibody, respectively, according to the manufacturer’s instructions (both from ThermoFisher, Grand Island, NY). Cells were also incubated with the corresponding phycoerythrin-labeled isotype control - IgG2a kappa for P-gp and IgG2b kappa for ABCG2 (both from ThermoFisher). P-gp or ABCG2 transporter activity was measured using rhodamine 123 or purpurin-18, respectively^[19]^. Cells were trypsinized and incubated for 30 min in complete medium (phenol red-free Richter’s medium with 10% FCS and penicillin/streptomycin) with the desired fluorescent substrate (0.5 µg/mL rhodamine 123 to detect P-gp or 15 µM purpurin-18 to detect ABCG2) in the presence or absence of 25 µM concentrations of the desired FIN or a positive control inhibitor (10 µM valspodar for P-gp or 10 µM fumitremorgin C for ABCG2) for 30 min at 37 °C in 5% CO_2_. Subsequently, cells were washed and incubated in substrate-free medium for 1 h at 37 °C continuing with or without inhibitor. Cells were subsequently analyzed with a FACSCanto flow cytometer (BD Biosciences, San Jose, CA) and data analysis was performed using FloJo v 10.4.2 (FlowJo LLC, Ashland, OR).

### ATPase assay

The ATPase assay was performed as described previously^[20]^. Total membrane vesicles were prepared from High Five insect cells overexpressing P-gp. The membranes were diluted with ATPase assay buffer (50 mM MES-Tris, pH 6.8 containing 50 mM KCl, 5 mM NaN_3_, 1 mM EGTA, 10 mM MgCl_2_, 2 mM DTT and 1 mM oubain) to reach a final concentration of 100 µg/mL and were incubated with the compounds at the noted concentrations for 10 min in the presence or absence of 0.3 mM sodium orthovanadate. The addition of 5 mM ATP (5 mM) started the reaction (20 min at 37 °C), after which SDS (2.5% final concentration) was added to terminate the reaction. The amount of inorganic phosphate released was quantified and the results were reported as a percentage of vanadate-sensitive ATPase activity with DMSO.

## RESULTS

### Generation and characterization of A673 cells that overexpress P-gp or ABCG2

**Figure 1:**
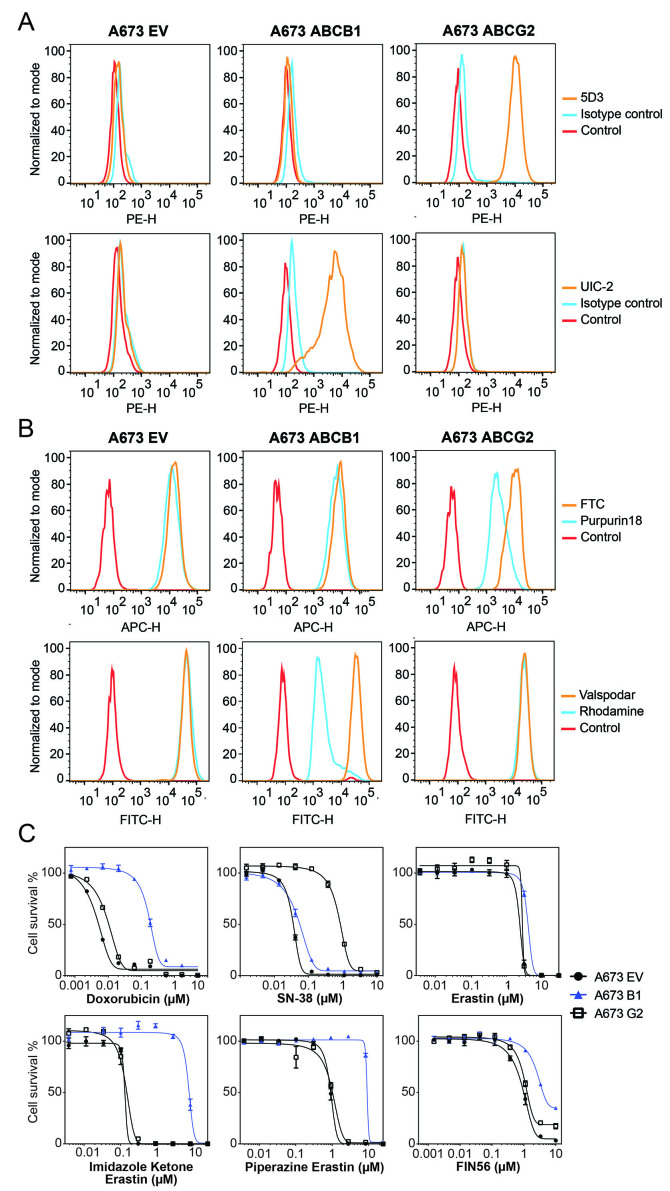
Characterization of A673 cells transfected to express P-gp or ABCG2. (A) Trypsinized A673 EV, B1 or G2 cells were incubated with 2% bovine serum albumin/PBS containing phycoerythrin-labeled antibody to detect ABCG2 (5D3) or P-gp (UIC-2), or the corresponding isotype control antibody for 20 min after which cells were washed in PBS. Control cells (no antibody) are denoted by red curves, isotype control staining is denoted by blue curves, and staining with specific transporter antibodies is denoted by orange curves (ABCG2 top row, P-gp bottom row). Results from one of three independent experiments are shown; (B) Trypsinized A673 EV, B1, and G2 cells were incubated with rhodamine 123 (0.5 µg/mL, for detection of P-gp) or purpurin-18 (15 µM, for detection of ABCG2) with or without appropriate inhibitor (10 µM valspodar for P-gp; 10 µM FTC for ABCG2) for 30 min, after which media was removed and replaced with substrate-free medium continuing with or without inhibitor for an additional 1 h. Cell autofluorescence (control) is denoted by red histograms, substrate efflux is denoted by blue histograms and cells with substrate and inhibitor are denoted by orange histograms. Results from one of three independent experiments are shown; (C) Three-day cytotoxicity assays were performed on A673 EV, B1, and G2 cells with doxorubicin, SN-38, erastin, imidazole ketone erastin, piperazine erastin and FIN56. Results from one of three independent experiments are shown and results are summarized in Table 1.

As the A673 cell line was reported to be sensitive to FINs^[4]^ and did not express P-gp or ABCG2, we transfected this cell line with either empty vector (A673 EV) or vectors containing the genes encoding human P-gp (A673 B1) or ABCG2 (A673 G2). We selected single clones with high levels of P-gp or ABCG2 based on measurement of antibody staining by flow cytometry and further characterized positive clones. As seen in Figure 1A, A673 B1 or A673 G2 cells were found to have much higher levels of the transporter proteins, as shown by increased staining with UIC-2 antibody or 5D3 antibody (orange histogram), respectively, compared to A673 EV cells which were negative for both transporters. P-gp-overexpressing MDR-19 cells and ABCG2-overexpressing R-5 cells served as positive controls for P-gp or ABCG2 surface expression, respectively (data not shown). Functional assays were also used to confirm transporter activity. A673 B1 cells readily transported rhodamine 123 and A673 G2 cells demonstrated increased purpurin-18 efflux, as shown by the left shift of the blue histogram for the two substrates, compared to empty vector-transfected cells (A673 EV), as illustrated in Figure 1B.

To verify that transporter levels were adequate to confer resistance to known substrates, we performed cytotoxicity assays with the P-gp substrate doxorubicin as well as the ABCG2 substrate SN-38. As seen in Figure 1C and Table 1, A673 B1 cells were resistant to doxorubicin, whereas A673 G2 cells exhibited little to no resistance to this compound. A673 G2 cells displayed increased resistance to SN-38. Having confirmed that the transfected cells expressed high levels of the desired transporters and that the transporters were indeed functional, we proceeded to use them to characterize the ability of P-gp and ABCG2 to confer resistance to FINs.

**Table 1:** Cross-resistance profile of transfected A673 cells*^a^*

**Compound**	**A673 EV GI_50_ (µM)**	**A673 B1 GI_50_ (µM)**	**RR**	**A673 G2 GI_50_ (µM)**	**RR**
SN-38	0.0018 ± 0.001	0.0024 ± 0.0004	1.3	0.042 ± 0.00013	23
Doxorubicin	0.010 ± 0.004	0.24 ± 0.075	24	0.017 ± 0.005	1.7
Erastin	2.2 ± 0.42	3.4 ± 1.2	1.5	1.9 ± 0.78	0.86
Erastin2	0.083 ± 0.004	0.17 ± 0.043	2	0.087 ± 0.024	1
Imidazole ketone erastin	0.31 ± 0.25	11.4 ± 5.4	38	0.33 ± 0.24	1.1
Piperazine erastin	1.6 ± 0.42	11 ± 2	6.9	1.4 ± 0.28	0.88
FIN56	0.70 ± 0.43	3.9 ± 2.8	5.6	0.99 ± 0.65	1.4
JKE-1674	1.0 ± 0.20	1.9 ± 0.18	1.9	2.7 ± 0.11	2.7
RSL3	0.055 ± 0.010	0.12 ± 0.008	2.2	0.06 ± 0.003	1.1
FinO_2_	2.8 ± 1.7	2.8 ± 1.7	1	3.0 ± 1.6	1.1
PACMA31	0.047 ± 0.056	0.07 ± 0.056	1.5	0.045 ± 0.039	0.96
ML-210	0.16 ± 0.031	0.33 ± 0.028	2.1	0.20 ± 0.037	1.2
GPX4 inhibitor 26a	0.077 ± 0.036	0.14 ± 0.050	1.8	0.093 ± 0.020	1.2
ML-162	0.095 ± 0.013	0.20 ± 0.057	2.1	0.12 ± 0.026	1.3
JKE-1716	1.4 ± 0.38	2.5 ± 0.43	1.8	0.81 ± 0.054	0.58

*^a^*Results presented are mean GI_50_ values +/- SEM (µM). Relative resistance (RR) value was determined by dividing the GI_50_ value for A673 cells expressing a transporter by the GI_50_ value for the A673 EV cells. Three independent experiments were performed.

### P-gp overexpression confers resistance to modified erastin derivatives

A673 EV, A673 B1 and A673 G2 cells were then used in 3-day cytotoxicity assays to determine whether the transporters could confer resistance to the FINs. While resistance due to the expression of ABCG2 or P-gp was not seen with most FINs examined, this was not true for some erastin derivatives that had been modified to improve water solubility. Overexpression of P-gp conferred relatively high levels of resistance to imidazole ketone erastin and piperazine erastin [Figure 1C], but we observed no resistance to erastin. FIN56 appeared to be a weak P-gp substrate, as A673 B1 cells were about 6-fold resistant [Table 1]. ABCG2 overexpression did not confer appreciable resistance to any of the FINs examined.

**Figure 2:**
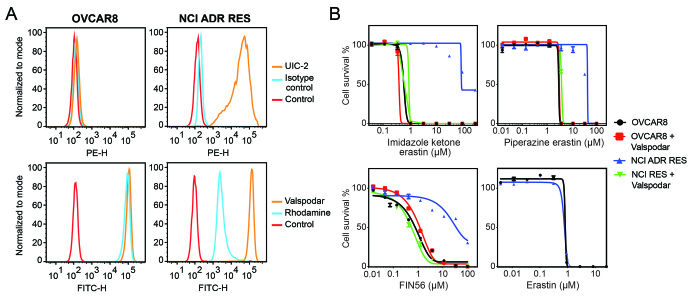
The OVCAR8 and NCI/ADR-RES cell line pair confirm P-gp substrates. (A) Trypsinized OVCAR8 or NCI/ADR-RES cells were incubated with 2% bovine serum albumin/PBS containing phycoerythrin-labeled UIC-2 antibody to isotype control antibody for 20 min, after which cells were washed in PBS and read on a flow cytometer. Control cells (no antibody) are denoted by red curves, isotype control staining is denoted by blue curves, and P-gp staining is denoted by orange curves (ABCG2 top row, P-gp bottom row). Results from one of three independent experiments are shown; (B) Three-day cytotoxicity assays were performed on OVCAR8 and NCI/ADR-RES cells with imidazole ketone erastin, piperazine erastin, FIN56 or erastin. Where noted, the P-gp inhibitor valspodar was added at a concentration of 10 µM. Results from one of three independent experiments are shown and results are summarized in Supplementary Table 1.

Since P-gp overexpression appeared to confer resistance to some FINs, we validated the results in parental OVCAR8 ovarian cancer cells and P-gp-overexpressing NCI/ADR-RES cells that were derived from OVCAR8 cells by selection with doxorubicin. As shown in Figure 2A, OVCAR8 cells do not express P-gp, as determined with the P-gp-specific monoclonal antibody UIC-2, while NCI/ADR-RES cells express high levels of the transporter, as shown by increased staining with the UIC-2 antibody in NCI/ADR-RES cells. Additionally, rhodamine efflux was observed in the NCI/ADR-RES cells, but not in the OVCAR8 cells. In this model system, when we performed cytotoxicity assays in the presence or absence of 10 µM valspodar, a P-gp inhibitor, we observed that P-gp overexpression conferred resistance to imidazole ketone erastin, piperazine erastin, and FIN56, and that valspodar reversed the resistance. P-gp overexpression in the NCI/ADR-RES line was not found to confer resistance to erastin or erastin2 [Figure 2B and Supplementary Table 1], in agreement with the results from the A673 cells.

### Deletion of ABCB1 in UO-31 cells increases sensitivity to erastin derivatives

**Figure 3:**
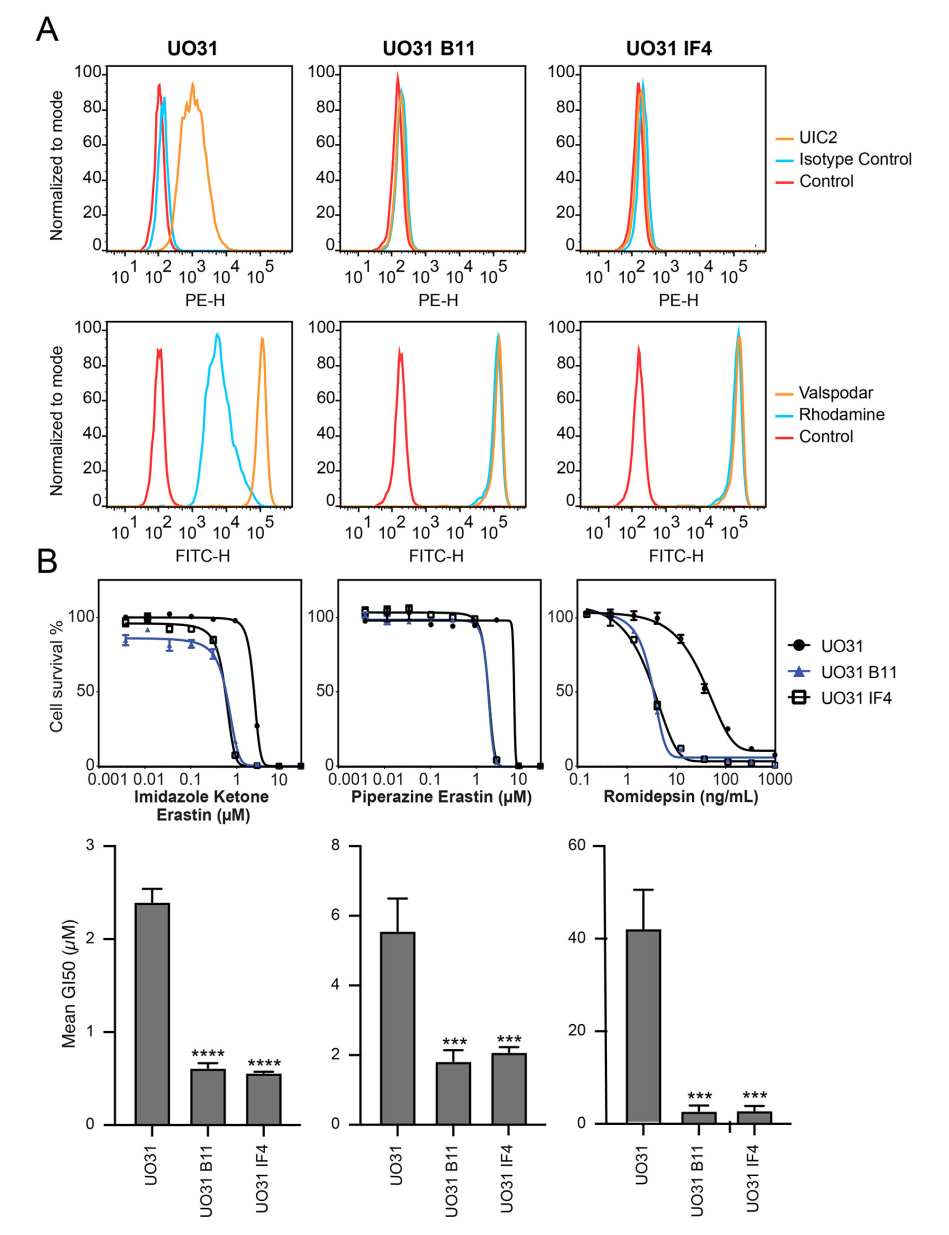
CRISPR-mediated deletion of *ABCB1* sensitizes UO-31 cells to FINs. (A) Top row: UO-31 cells or the *ABCB1* knockout clones (B11, 1F4) were trypsinized and incubated with 2% bovine serum albumin/PBS containing phycoerythrin-labeled UIC-2 antibody or isotype control antibody for 20 min after which cells were washed in PBS and read on a flow cytometer. Control cells (no antibody) are denoted by red curves, isotype control staining is denoted by blue curves, and staining with UIC-2 is denoted by orange curves. Bottom row: Cells were incubated with rhodamine 123 (0.5 µg/mL) with or without 10 µM valspodar for 30 min, after which media was removed and replaced with substrate-free medium continuing with or without inhibitor for an additional 1 h. Cell autofluorescence (control) is denoted by red histograms, rhodamine efflux by blue histograms, and cells with rhodamine and inhibitor are denoted by orange histograms. Results from one of three independent experiments are shown; (B) Three-day cytotoxicity assays were performed on UO-31 cells or the *ABCB1* knockout clones with romidepsin, imidazole ketone erastin or piperazine erastin. Results from one of three independent experiments are shown. GI_50_ values from 3 independent experiments are shown under the representative graphs. Significance was determined by a one-way ANOVA followed by a Dunnett test for multiple comparisons. Asterisks denote significant differences from the parental UO-31 cell line, where ***P < 0.001 or ****P < 0.0001.

The renal carcinoma cell line UO-31 is known to have detectable levels of P-gp and displays rhodamine efflux^[21]^. To determine if the expression of P-gp in this cell line is high enough to confer resistance to imidazole ketone erastin or piperazine erastin, two of the best substrates for P-gp, we performed CRISPR-mediated deletion of *ABCB1* and selected two clones that had lost expression. As shown in Figure 3A, while UO-31 cells do stain positively with the UIC-2 antibody, as shown by increased staining with the UIC-2 antibody (orange histogram), the two knockout clones, B11 and 1F4, no longer react with the antibody, as detected by flow cytometry. Additionally, we found that the knockout clones no longer efflux the P-gp substrate rhodamine 123, as shown by increased intracellular fluorescence of rhodamine 123 (blue histogram) in the clones, suggesting that the *ABCB1* gene had been deleted. The knockout clones also demonstrate increased sensitivity to the P-gp substrate romidepsin [Figure 3B]. When we performed cytotoxicity assays with imidazole ketone erastin and piperazine erastin, the knockout clones displayed increased sensitivity to both compounds by about 3- to 4-fold compared to parental cells; however, no difference in sensitivity to erastin was noted [Supplementary Table 2]. Similar results were obtained when we performed cytotoxicity assays with imidazole ketone erastin and pierazine erastin in the presence of 10 µM of the P-gp inhibitor valspodar [Supplementary Tables 1 and 3]. Thus, even relatively low levels of P-gp may cause resistance to the erastin analogs.

### Ferroptosis inducers stimulate the ATPase activity of P-gp

**Figure 4:**
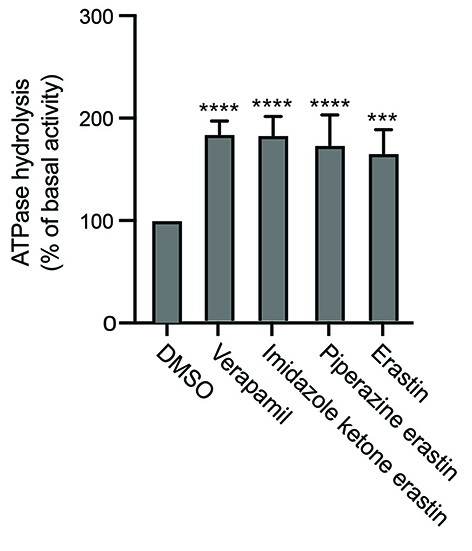
Effect of erastin derivatives on ATPase activity of P-gp. The effect of imidazole ketone erastin, piperazine erastin and erastin or the vanadate-sensitive ATPase activity of P-gp was determined as outlined in Materials and Methods. Basal ATPase activity was compared to that in the presence of 10 µM concentrations of the compounds; verapamil at 10 µM served as a positive control for stimulation of ATPase activity. Significance was determined by a one-way ANOVA followed by a Dunnett test for multiple comparisons. Asterisks denote significant differences from the DMSO control, where ***P < 0.001 or ****P < 0.0001.

The effect of erastin, imidazole ketone erastin, and piperazine erastin on the ATPase activity of P-gp was subsequently examined. While several P-gp substrates and some inhibitors have been shown to stimulate the ATPase activity of P-gp, not all substrates do so. We found that all three of the compounds stimulated the ATPase activity of P-gp to a degree comparable to that of verapamil, which is used as a positive control [Figure 4]. The ATPase stimulation serves as a confirmation of the interaction between imidazole ketone erastin and piperazine erastin and P-gp, given the close connection between ATP hydrolysis and substrate efflux^[22]^. While P-gp does not appear to confer resistance to erastin in our studies, erastin does stimulate ATPase activity. This suggests that erastin interacts with P-gp, but likely has a slow off rate, potentially acting more as a weak inhibitor than a substrate.

### Ferroptosis inducers inhibit P-gp- and ABCG2-mediated transport

**Figure 5:**
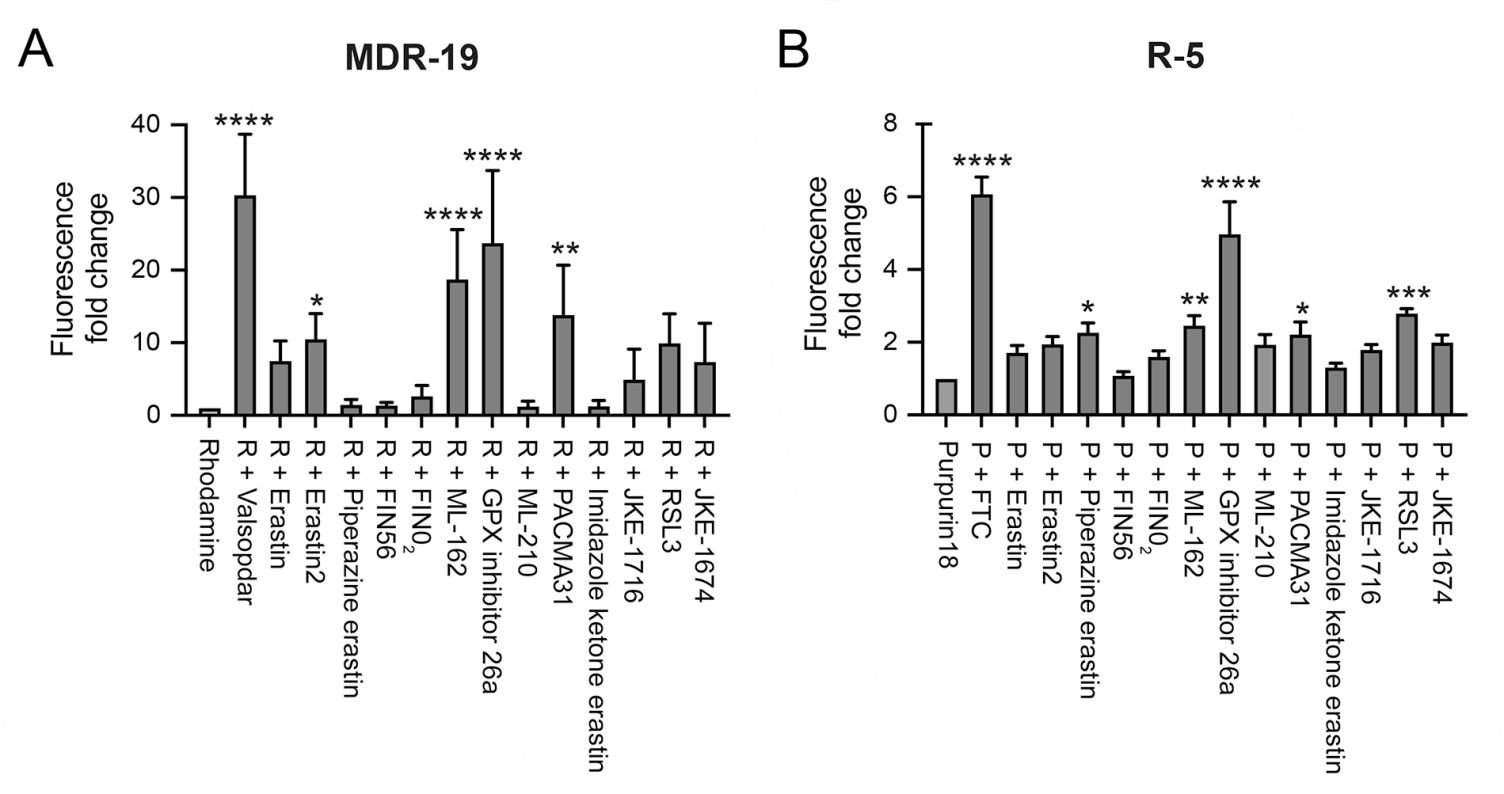
FINs inhibit P-gp and ABCG2 transport activity. P-gp-overexpressing MDR-19 cells (A) or ABCG2-overexpressing R-5 cells (B) were incubated with 0.5 µg/mL rhodamine 123 or 15 µM purpurin-18, respectively, in the absence or presence of specific inhibitor (10 µM valspodar for P-gp and 10 µM FTC for ABCG2) or 10 µM concentrations of the FINs for 30 min after which the medium was removed and replaced with substrate-free medium with or without the inhibitor. Cells were then incubated for an additional 1 h. Inhibition of P-gp or ABCG2 was determined by calculating the fold increase in intracellular fluorescence, with fluorescence levels in cells incubated with rhodamine or purpurin-18 alone assigned a value of 1. Significance was determined from three independent experiments using a one-way ANOVA followed by a Dunnett test for multiple comparisons. Asterisks denote significant difference from the rhodamine or purpurin-18 control, where *P < 0.05, **P < 0.01, ***P < 0.001, or ****P < 0.0001. P: Purpurin-18; R: rhodamine.

As targeted therapies are known to act as inhibitors of ABC transporters, we next characterized the ability of the FINs to act as inhibitors of P-gp or ABCG2. At a concentration of 10 µM, erastin2, ML-162, GPX4 inhibitor 26a, and PACMA31 inhibited P-gp-mediated rhodamine 123 transport, resulting in a 5- to 10-fold increase in rhodamine fluorescence in MDR-19 cells [Figure 5A]. In ABCG2-expressing R-5 cells, GPX inhibitor 26a had the greatest effect on purpurin-18 efflux, while piperazine erastin, ML-162, PACMA31 and RSL3 also significantly inhibited purpurin-18 efflux [Figure 5B]. These results suggest that drug-drug interactions might occur during treatment with FINs.

## DISCUSSION

Ferroptosis induction by small molecules is a novel way to induce cell death in cancer cells and several cancer cell types are sensitive to ferroptosis induction, such as renal cell carcinoma, diffuse large B-cell lymphomas, as well as many chemotherapy-resistant cancer subtypes^[4,23]^. FINs are effective in xenograft mouse models and have been suggested as potential cancer treatments^[6]^. Despite the recent proliferation of papers describing novel molecules that can induce ferroptosis^[9-11,24]^, very few studies have addressed potential interactions with ABC transporters that might limit bioavailability or brain penetration. In our study, we found that the FINs FIN56, imidazole ketone erastin and piperazine erastin are transported by P-gp, suggesting that their oral bioavailability and/or brain penetration may be compromised. Additionally, we found that the FINs ML-162, GPX inhibitor 26a, and PACMA31 act as inhibitors of P-gp. Interestingly, the most potent P-gp inhibitor, GPX inhibitor 26a, was also the most potent ABCG2 inhibitor, suggesting that treatment with FINs may cause drug-drug interactions.

Our findings regarding erastin differ from those of Zhou *et al*. who reported erastin as a P-gp substrate^[25]^. However, we note that they used a P-gp-expressing cell line that was generated by gradually increasing exposure to paclitaxel^[25]^. While selection with paclitaxel can lead to P-gp overexpression, other mechanisms of resistance can arise^[26]^. It cannot be ruled out that other mechanisms besides efflux by P-gp may have caused the resistance to erastin observed by Zhou *et al*. Unfortunately, they did not perform cytotoxicity assays in the presence of a P-gp inhibitor, which would have confirmed the role of P-gp. In contrast, we did not observe erastin resistance in cells that were transfected to express P-gp without selection with an anticancer drug, and this result was confirmed in a selected cell line. We thus conclude that erastin is not a P-gp substrate and that the cell line used by Zhou *et al*. may have another mechanism at work that can confer resistance to erastin^[25]^.

It is not surprising that the FINs, which are essentially targeted therapies, interact with drug transporters, as many targeted therapies have been shown to either be substrates or inhibitors of P-gp or ABCG2^[27]^. The BCR-ABL inhibitors imatinib, nilotinib, dasatinib, and bosutinib have been found to be substrates of P-gp and ABCG2 at low concentrations, while they act as inhibitors of the proteins at higher concentrations^[28-31]^. Overexpression of *ABCB1* has been demonstrated in tumor samples obtained from patients whose tumors have developed resistance to the ALK inhibitor ceritinib in the absence of secondary ALK mutations^[32]^. Ceritinib has also been reported to inhibit P-gp- and ABCG2-mediated transport^[33]^. Additionally, both P-gp and ABCG2 have been shown to confer resistance to several structurally different aurora kinase inhibitors^[34,35]^.

The ability of P-gp and ABCG2 to affect brain penetration of targeted therapies has most dramatically been demonstrated in mouse models in which the *ABCB1* homologs *Abcb1a* and *Abcb1b* are knocked out, the ABCG2 homolog *Abcg2* is knocked out, or all of the homologous transporters have been deleted. Brain concentrations of the Janus kinase 1/2 inhibitor momelotinib 24 h after oral administration were 6.5-fold, 3-fold and 48-fold higher in mice deficient in Abcg2, Abcb1a/b, or Abcg2;Abcb1a/b, respectively, compared to control mice^[36]^. Similarly, 24 h after oral administration of the BCR-ABL inhibitor ponatinib, brain concentrations were 2.2-fold, 1.9-fold and 25.5-fold higher in mice deficient in Abcg2, Abcb1a/b, or Abcg2;Abcb1a/b, respectively, compared to wild-type controls^[37]^. Thus, P-gp and ABCG2 can have a profound effect of limiting brain penetration of targeted therapies that are substrates of the transporters. Brain accumulation or oral bioavailability of FIN56, imidazole ketone erastin or piperazine erastin could similarly be affected, as they were found to be transported by P-gp.

In conclusion, we have demonstrated that the FINs FIN56, imidazole ketone erastin, and piperazine erastin are substrates of P-gp, suggesting a potential reduction in oral bioavailability and brain penetration. ML-162, GPX inhibitor 26a and PACMA31 were found to inhibit the transport activity of P-gp and/or ABCG2, suggesting potential drug-drug interactions. Thus, our findings will be valuable as these compounds are pursued clinically.

## DECLARATIONS

### Availability of data and materials

The authors declare that all the data supporting the findings of this study are available within the paper and its Supplemental Data.

### Copyright

© The Author(s) 2023.
