# Supplementary material for: The multidrug resistance transporter P-glycoprotein confers resistance to ferroptosis inducers
Source: Cancer Drug Resist. 2023 Jul 27;6(6):468–80. doi: 10.20517/cdr.2023.29 (PMC10571053; doi:10.20517/cdr.2023.29)

Supplementary Data for:
The Multidrug Resistance Transporter P-glycoprotein Confers Resistance to Ferroptosis Inducers

William J. E. Frye, Lyn M. Huff, José M. González Dalmasy, Paula Salazar, Rachel M. Carter, Ryan T. Gensler, Dominic Esposito, Robert W. Robey, Suresh V. Ambudkar, Michael M. Gottesman

Supplementary Table 1. Cross resistance profile of OVCAR8 and NCI/ADR-RES cells with ferroptosis inducers

Compound	n	OVCAR8 GI_{50}	NCI ADR RES GI_{50}	OVCAR8 + Inhibitor GI_{50}	NCI ADR RES + Inhibitor GI_{50}
Erastin	3	2.13±1.2	1.94±1.2	NA	NA
Erastin2	3	0.136±0.12	0.144±0.085	NA	NA
Imidazole Ketone Erastin	3	0.361±0.19	84.6±1.7	0.301±0.082	0.545±0.30
Piperazine Erastin	3	1.92±0.90	34.6±10	3.07±0.066	3.72±0.18
Fin56	3	0.527±0.34	51.4±23	0.977±0.63	0.320±0.25
JKE-1674	3	1.25±0.73	0.85±0.44	NA	NA
RSL3	3	0.0760±0.044	0.0580±0.017	NA	NA
Fin02	3	1.54±0.62	0.88±0.13	NA	NA
PACMA31	3	0.556±0.68	0.776±0.81	NA	NA
ML-210	3	0.177±0.087	0.108±0.029	NA	NA
GPX4 Inhib 26a	3	0.192±0.13	0.151±0.085	NA	NA
ML-162	3	0.202±0.038	0.175±0.093	NA	NA
JKE-1716	3	10.6±5.9	8.37±.93	NA	NA

Results presented are mean GI_{50} values +/- SEM. Three independent experiments were performed. Concentrations are in μM.

`

Supplementary Table 2. Cross resistance profile of UO-31 cells and *ABCB1*-knockout clones

Compound	n	UO31	UO31 B11	UO31 IF4
Erastin (µM)	3	0.334±0.06	0.614±0.0050	0.552±0.10
Romidepsin (ng/ml)	3	41.5±8.07	2.77±1.16	3.01±0.91
Imidazole Ketone Erastin (µM)	3	2.40±0.14	0.617±0.05	0.563±0.01
Piperazine Erastin (µM)	3	5.57±0.93	1.83±0.31	2.09±0.14

Results presented are mean GI_{50} values +/- SEM. Three independent experiments were performed. Concentrations are in µM.

Supplementary Table 3. UO-31 Cells with and without P-gp inhibitor valspodar

Drug	n	UO31	UO31 + Valspodar
Imidazole Ketone Erastin	3	1.87±0.055	0.289±0.0090
Piperazine Erastin	3	3.06±0.10	.97±0.0095

Results presented are mean GI_{50} values +/- SEM. Three independent experiments were performed. Concentrations are in µM.

Supplementary Figure 1. UO-31 Cells with and without the P-gp inhibitor valspodar

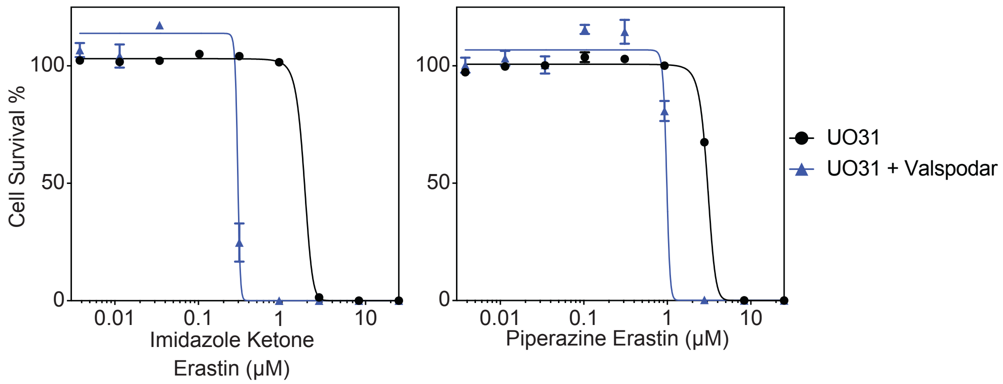

Supplement: Supplementary file 1 [file cdr-6-3-468-SupplementaryMaterials.pdf]
